# Myths about Intimate Partner Violence and Moral Disengagement: An Analysis of Sociocultural Dimensions Sustaining Violence against Women

**DOI:** 10.3390/ijerph17218139

**Published:** 2020-11-04

**Authors:** Chiara Rollero, Norma De Piccoli

**Affiliations:** Department of Psychology, University of Turin, 10124 Torino, Italy; norma.depiccoli@unito.it

**Keywords:** intimate partner violence, sociocultural dimensions, ambivalent sexism, lay theories of gender

## Abstract

Intimate partner violence (IPV) is a public health issue worldwide and a serious violation of human rights. Recognizing IPV as a form of violence is essential for both victims who need help and offenders who can join treatment programs. Furthermore, only a society able to identify violence can effectively deal with IPV. The present study is aimed at investigating the role of sociocultural dimensions (i.e., ambivalent sexism toward women, ambivalence toward men, and lay theories about gender differences) in sustaining myths about IPV and moral disengagement. The participants were 359 university students (76.5% female). The results show that hostile sexism toward women plays a key role in sustaining both myths and moral disengagement. Moreover, benevolence toward men and biological lay gender theories (i.e., “naïve” theories assuming that sex differences are a product of biology and genetics) significantly affected the endorsement of IPV myths. The implications are discussed.

## 1. Introduction

Intimate partner violence (IPV) represents a public health issue worldwide, as well as a gross violation of human rights. It takes several forms, such as physical and sexual violence, emotional and social abuse, threats and intimidation, and economic deprivation [1]. It involves not only the victim and the perpetrator, but the society as well, because the way in which IPV is perceived and the attitudes held by the society can lead to several damaging consequences, such as the isolation of the victims, the inaction of professionals, and the transmission of harmful norms among peers [2,3]. Indeed, recognizing abusive behaviors between intimates as a form of violence is essential at the individual level, for both victims who need to receive help and offenders who can join treatment programs, and at the societal and community level, because an environment able to identify violence can effectively deal with IPV [2,3]. Although less studied than individual variables, sociocultural dimensions sustaining IPV need to be addressed. As Waltermaurer has argued, “in a community where a higher proportion of the general population feels that IPV is justifiable, a potential perpetrator will be more likely to feel he or she has the right, should the case arise” [3] (p. 167).

There are numerous biases that influence the perception of IPV allowing the justification of violence, lessening claims of behaviors, or suggesting that only specific types of women are abused [4]. The literature refers to such biases as myths, defined as false beliefs about violence that promote blaming the victim and exonerating the offender [5]. Specifically, domestic violence myths are “stereotypical beliefs that are generally false but are widely and persistently held, and which serve to minimize, deny, or justify physical aggression against intimate partners” [6] (p. 5). In the past 40 years, those beliefs that blatantly blame women have become less publicly tolerable and acceptable. However, many of the myths that the victims did something to cause the abuse and the perpetrator is not completely at fault are still present, although in more subtle expressions [2]. Furthermore, domestic violence myth acceptance is associated with a set of belief systems at work in the maintenance of the social hierarchy between genders [7].

Two main theoretical perspectives have analyzed violence myth acceptance: the feminist approach and the defensive attribution theory. According to the feminist approach [8,9], IPV is rooted in the patriarchy, as social relationships between men and women are organized hierarchically. According to this perspective, IPV is a consequence of gender inequality and the myths legitimizing violence sustain patriarchal abuse against women [5,10].

The defensive attribution theory argues that individuals blame the victim “in order to defend themselves against the idea of enduring (harm avoidance) or causing harm (blame avoidance)” [7, p. 239]. This is also an expression of the cognitive necessity of the human being to believe that events happen according to a principle of justice, as the just world theory has clearly shown [11]. In other words, people need to see the victim as the recipient of just reward.

These two theoretical perspectives can be seen as complementary in explaining myths about IPV, as individual cognitive processes may be reinforced within the social and cultural environment, which in turn is influenced by individuals’ attitudes and perceptions.

### 1.1. Sociocultural Dimensions and IPV: The Role of Sexism and Lay Gender Theories

The literature has largely demonstrated that violence myths are strongly related to the endorsement of gender stereotypes and ambivalent sexist attitudes. According to the Ambivalent Sexism Theory [12,13], gender stereotypes comprise significant ambivalence by one sex toward the other and may be conceptualized through four related dimensions. In reference to women, hostile sexism (HS) represents an adversarial perception of gender relations in which women are seen as seeking to control men and usurping their power, whereas benevolent sexism (BS) idealizes women as pure persons who should be protected and supported, but it implies that they are frail and best suited for conventional gender roles. Similarly, stereotypes toward men include both hostility toward men (HM) and benevolence toward men (BM). The first conveys hostility toward male dominance and the ways men make use of control within intimate relationships. Benevolence toward men encompasses positive attitudes rooted in appreciation for men’s role as providers and protectors.

Research based on the Ambivalent Sexism Theory found that individuals who endorse sexist attitudes show higher myth acceptance than those who do not, wherein both students and adults with a more traditional perception of gender roles are more likely to blame the victim for the abuse than those who hold more non-traditional conceptions, e.g., [14,15,16]. In a recent study with university students, Rollero and Tartaglia [17] found that two dimensions of ambivalent sexism are particularly predictive of violence myths, e.g., hostility toward women and benevolence toward men. They both contribute to legitimizing partner violence and this, in turn, leads to undervaluing the seriousness of the abuse [18].

Such stereotypical attitudes are strictly related to lay theories of gender. Lay theories are rooted in the theoretical tradition of individuals as ‘‘naıve scientists’’ [19], who hold specific theories about social groups and utilize them to guide their perceptions of such groups, comprising their own in-group [20]. In reference to men and women, one of the main focuses of such theories deals with the nature versus nurture debate about the origin of gender differences. Several scholars [20,21,22] converged to the presence of two prevalent lay theories of gender, i.e., a biological gender theory and a social gender theory. The first contends that sex differences are a product of biology and genetics and imply essentialistic attributes associated with the sexes [22,23]. By contrast, the social approach claims that sex differences derive mainly from the contrasting social positions of men and women and stresses the power of the context and social practices in shaping gender differences [20]. In this perspective, gender differences are considered as results of social constructions.

Research has demonstrated that the biological theory is associated with higher levels of gender stereotypes [23], self-stereotyping [20], endorsement of gender discriminatory practices [24], and higher justification of the current gender system [25], among both adults and university students. For instance, in their studies involving university students in Australia and the UK, Morton and colleagues [24] found that exposure to essentialist theories increased men’s and women’s acceptance of gender inequality and boys’ support for discriminatory practices. Furthermore, a recent experimental study involving college students found that participants exposed to social constructionist explanations of gender differences showed a greater recognition of discriminatory behavior than those exposed to biological explanations or no explanation [22].

### 1.2. Moral Disengagement in Violence and Harassment toward Women

The social cognitive theory of moral disengagement [26] explains how people self-regulate a diverse range of morally transgressive behaviors. By means of eight psychosocial mechanisms, anticipatory self-sanctions can be disengaged from immoral conduct by cognitively restructuring the harmful behavior (i.e., “moral justification”, “euphemistic labelling”, and “advantageous comparison”), masking causal agency (i.e., “displacement of responsibility” and “diffusion of responsibility”), disregarding or misrepresenting injurious consequences (i.e., “distortion of consequences”), and denigrating the victims (i.e., “attribution of blame” and “dehumanization”) [27].

Recently, this perspective has been applied to sexual harassment toward women [28,29]. Sexual harassment refers to unwelcome social-sexual misconduct (e.g., sexist jokes, sexual epithets, displays of pornography) that occurs due to the target’s sex and is sufficiently pervasive to negatively alter the conditions of the victim, creating an intimidating, hostile, and abusive work/study context [30,31].

In the domain of sexual harassment, for example, euphemistic labelling allows harassing behaviors to be renamed as “flirting”, “banter”, or “joking”, thereby masking their harmful appearance. Another example is when the perceived damaging consequences of harassment are distorted and seen as being pleasurable or gratifying for the target [28]. To date, very few empirical studies have examined the specific role of moral disengagement on sexual harassment. However, when the association between moral disengagement and men’s self-reported harassment proclivity was investigated, the findings from university students in the UK showed that moral disengagement had an indirect effect in affecting men’s tendency to harass by lowering their moral judgment and negative affect about the harassment, conversely intensifying positive affect [29].

Although distinct, sexual harassment and IPV represent two specific forms of violence that share some characteristics, such as unwanted conduct toward the victim, minimized effects, and likelihood not to be recognized as abuse [17,28]. Furthermore, we argue that they are affected by the same sociocultural dimensions. However, to our best knowledge, the role of gender stereotypes and lay gender theories in moral disengagement toward harassment has not been investigated yet.

### 1.3. The Current Study

The present study aims at investigating the role of sociocultural dimensions (i.e., ambivalent sexism toward women, ambivalence toward men, and lay theories about gender differences) in sustaining myths about IPV and moral disengagement in sexual harassment.

Based on previous research, we hypothesized that hostile sexism toward women and benevolence toward men would be positively associated with domestic violence myth acceptance [17,18]. Since no previous study has investigated the role of lay gender theories on IPV myth acceptance, we aimed at investigating whether lay theories would influence IPV myth acceptance beyond sexist attitudes. We supposed that the biological theory of gender would be positively associated with domestic violence myth acceptance, and the social theory would be negatively associated with domestic violence myth acceptance, in line with studies on lay theories and gender discrimination [23,24,25].

Since we argue that moral disengagement toward sexual harassment and domestic violence myths share the same sociocultural roots, in line with hypotheses concerning IPV, we supposed that moral disengagement would be positively influenced by HS, BM, and the biological theory of gender, and negatively by the social theory of gender.

## 2. Materials and Methods

### 2.1. Participants

The study enrolled 359 students (76.5% women), whose mean age was 21.18 years (age range = 18–34, SD = 2.57) attending the University of Turin, Italy.

Among them, 39.1% were students in Psychology, 18% in Arts and Humanities; 12.6% in Engineering, 8.9% in Law, 5.3% in Educational Sciences, 4.5% in Medicine, 3.9% in Information Technology, 2.8% in Biology and Chemistry, and 2.2% in Economics.

Concerning nationality, 95.8% were Italians, 3.4% had other citizenships, and 0.8% had dual citizenship. Most of them (65.5%) lived with their family, 17.4% with friends or roommates, 7.6% alone, 6.7% with the partner, and 2.8% in a university residence. For sexual orientation, 87.4% stated being exclusively or mainly heterosexual, 4.8% exclusively or mainly homosexual, and 3.9% bisexual.

### 2.2. Procedure and Measures

The Ethics Committee of the University of Turin, Italy, approved the study protocol (CERP 131,118–3 May 2020). Participants were recruited via students’ assistance during classes. Specifically, trained students attending the last year of the Faculty of Psychology recruited participants using snowball sampling techniques. Subjects were informed that their participation was voluntary and anonymity was granted. No compensation was given for their enrollment.

Data were collected by means of a self-report questionnaire, which took approximately 20 min to complete.

When available, we used validated scales in the Italian language. Otherwise, we translated and back-translated scales from English to Italian using the back-translation methodology.

The questionnaire included the following measures:

1. The short version of the Ambivalent Sexism Inventory [11,32] including 12 items measuring hostile sexism toward women (6 items, e.g., “Once a woman gets a man to commit to her, she usually tries to put him on a tight leash”, Cronbach’s α = 0.87) and benevolent sexism toward women (6 items, e.g., “Women should be cherished and protected by men”, Cronbach’s α = 0.84). The items were rated on a 6-point point Likert-type scale ranging from “strongly disagree” (0) to “strongly agree” (5). The Italian validated version of this scale was used [32].
2. The short version of the Ambivalence Toward Men Inventory [12,32] measuring hostile sexism toward men (6 items, e.g., “Men will always fight to have greater control in society than women”, Cronbach’s α = 0.74) and benevolent sexism toward men (6 items, e.g., “Men are more willing to put themselves in danger to protect others”, Cronbach’s α = 0.79). The items were rated on a 6-point point Likert-type scale ranging from “strongly disagree” (0) to “strongly agree” (5). The Italian validated version of this scale was used [32].
3. The Gender Theory Questionnaire [20] assessing lay theories about gender differences. It included two subscales, one measuring participants’ endorsement of a biological theory of gender (5 items, e.g., “When men and women differ in some way, it is likely that the difference is due to biological factors”, Cronbach’s α =0.79), and the other measuring participants’ endorsement of a social theory of gender (6 items, e.g., “If social situations change, the characteristics we attribute to gender categories will change as well”, Cronbach’s α = 0.73). Items were rated on rated on a 6-point scale ranging from “strongly disagree” (1) to “strongly agree” (6). For this scale, we followed the back-translation methodology.
4. The Domestic Violence Myth Acceptance Scale [6] including 18 items (Cronbach’s α = 0.84) measuring participants’ endorsement of domestic violence myths (e.g., “A lot of domestic violence occurs because women keep on arguing about things with their partners”). The items were rated on a 7-point point Likert-type scale ranging from “strongly disagree” (1) to “strongly agree” (7). For this scale, we followed the back-translation methodology. The short version of the Moral Disengagement in Sexual Harassment Scale [28,29] including 8 items (Cronbach’s α = 0.82) assessing moral disengagement toward harassment (e.g., “In a workplace/University place with a relaxed atmosphere, men cannot be blamed for “trying it” with attractive women when they get the chance”). The items were rated on a 7-point point Likert-type scale ranging from “strongly disagree” (1) to “strongly agree” (7). For this scale, we followed the back-translation methodology.
5. A list of socio-demographic items, including gender, age (i.e., “year of birth”), faculty (i.e., “what faculty do you attend?”), and nationality (i.e., “what is your nationality?”).

### 2.3. Data Analyses

After bivariate descriptive statistics, we carried out two hierarchical regression analyses to test our hypotheses. Specifically, in the first step, we entered sex as a predictor of domestic violence myth acceptance and moral disengagement. In the second step, we entered BS, HS, BM, HM. Finally, in the third step, we entered biological and social theory of gender.

All statistical analyses were carried out using IBM SPSS Statistics version 26.0 software (IBM, Armonk, NY, USA).

## 3. Results

### 3.1. Bivariate Analyses

T-tests were carried out to assess gender differences on the study variables. As shown in Table 1, men outscored women on HS, BM, moral disengagement, as well as on the endorsement of biological theory of gender and myths about domestic violence. On the contrary, women reported higher levels of HM and endorsement of social theory of gender. No significant gender differences emerged in relation to BS.

Zero-order correlations between scales were performed. As shown in Table 2, domestic violence myth acceptance was positively related to the four dimensions of ambivalent sexism, biological theory of gender, and moral disengagement, and negatively related to social theory of gender. Similarly, moral disengagement was positively linked to BS, HM, BM, and biological theory, and negatively linked to social theory. Finally, all the dimensions of ambivalent sexism were positively correlated with each other.

### 3.2. Regression Analyses

Two hierarchical regression analyses were performed to predict, respectively, domestic violence myth acceptance and moral disengagement in sexual harassment.

Concerning myth acceptance, as reported in Table 3, the model was significant and explained a good proportion of the dependent variable. Specifically, HS and BM were the strongest predictors of the endorsement of domestic violence myths. When lay theories were inserted, the biological perspective was significant, although it did not significantly increase the explained variance of violence myth acceptance.

As seen in Table 4, even the model about moral disengagement was significant and highly predicting. Gender, i.e., being male, was associated with moral disengagement in the first step and remained significant in the final model. As for myth acceptance, HS played a relevant role, whereas lay theories did not significantly increase the explained variance of the dependent variable.

## 4. Discussion

The research here presented was aimed at assessing sociocultural dimensions related to myths about IPV and moral disengagement in sexual harassment. Violence against women is not just an individual or intimate relationship issue. It involves social and cultural attitudes that may help in recognizing the abuse or legitimizing the perpetrator and blaming the victim.

Findings about gender differences reveal that men and women reported different scores on most of the scales, except for BS. Indeed, in line with previous research on gender stereotypes involving both adults and university students [12,13,17,33], the men were more likely to endorse hostile attitudes toward women (HS), benevolent attitudes toward their in-group (BM), and the biological theory of gender, whereas women showed higher hostility toward men (HM). Male participants also obtained higher scores on IPV myths and moral disengagement.

However, when the effect of other variables was concurrently tested by means of a regression model, gender remained significant only for moral disengagement. This may have been due to the specificity of the outcome, as harassment may be perceived as less serious and severe than intimate partner violence. In this sense, both men and women recognize myths endorsing IPV, but the men can be less able to identify violation of moral norms in the case of harassment. In other words, if the perception of IPV is similar across genders, the women may be more sensitive than the men to detect harassment behaviors, because they are likely to have experienced such actions as victims.

Hostile sexist attitudes toward women were the most powerful predictor of both IPV myths and moral disengagement, and benevolence toward men was positively related to IPV myths as well. This is consistent with previous studies carried out with university students, which found that sexist individuals show a higher myth acceptance than non-sexist individuals, because their conception of gender roles allows them to blame the victim for the sexual assault and justify the offender [17,18,34]. Notably, it is benevolence toward the perpetrators’ group, i.e., men, which fosters beliefs legitimizing IPV, and it is hostility toward the victims’ group, i.e., women, which facilitates both such beliefs and the justification of morally transgressive behaviors. These findings support the conception of gender violence as a culture-rooted phenomenon, in line with Willie and Kershaw [35], who proposed a social-ecological model to address IPV and other forms of abuse. In this framework, risk factors for victims and perpetrators are conceptualized not only at the relationship and individual level, but also at the societal and community level [35,36]. The few ecological analyses on this topic supported this model. The authors recently investigated the relation between USA state-level Gender Inequality Index values and prevalence of lifetime IPV, showing that the Gender Inequality Index was positively associated with the prevalence of any form of gender violence across states [35]. Similarly, in a study involving Spanish women, Redding and colleagues [37] showed that greater gender inequality was linked to higher rates of IPV mortality.

Our findings for the role of sexist attitudes are consistent with research about perpetrators. Denial of personal responsibility, victim blaming or other external attributions are frequent psychological processes among offenders, who are also inclined to justify their behaviors [38]. In a recent study carried out with perpetrators of IPV in treatment, the participants stated that they were socialized to the prescriptive dimension of gender, that requires men to be agentic, authoritarian and assertive, and this had a significant impact on their conception of intimate relationships and gender roles [39]. The men involved in that study claimed that reasoning about their sexist attitudes and taking moral responsibility for their violence were the trigger to reinstate moral agency, which in turn allowed them to engage in positive changes [39].

Finally, our results partially support the relationship between some lay theories of gender and IPV myths. Individuals who adopt an essentialistic perspective, based on the idea that sex differences are a product of biology and genetics, are more prone to justifying gender violence. To our knowledge, this was the first attempt to explore the impact of lay theories on IPV myths and moral disengagement. However, the present findings are consistent with research demonstrating the association between the biological theory and higher levels of gender stereotypes, gender discriminatory practices, and higher justification of the current gender system, even among University students [22,24,25]. However, the impact of biological theory slightly increased the variance explained. Furthermore, contrary to our expectations, the social theory of gender was not related either to IPV myths or to moral disengagement. This may be due to the specific population, i.e., university students, who are similar for educational level and age, and thus may share similar conceptions of cultural influences on gender roles. Anyway, we argue that the role of lay theories should be addressed in more depth, as such theories are a key variable in gender relationships, strictly related to the possibility of fostering change. Indeed, if people believe that some characteristics are biologically determined, changing them and the associated social roles may not be an option. On the contrary, when the origin of a characteristic is conceived as rooted in culture and socialization processes, such a trait can be seen as changeable and flexible [40].

The present paper presents some limitations which suggest directions for future research. First, participants were university students with a relatively high educational level and a young age. Future research should explore the same issues in people with other characteristics, that may have an impact on gender stereotypes and attitudes toward violence and harassment. Moreover, the sample size was not so large, and we used self-report measures, which may be affected by social desirability, especially considering gender attitudes. Snowball sampling design was also a limitation, as it did not imply random selection and thus did not allow representativeness.

Furthermore, all participants recruited for this study came from Italy, one of the least egalitarian countries in Europe in terms of gender equality [41], where gender stereotypes are more intense than in in the other Western countries [32,42]. According to the European Union Agency for Fundamental Rights [43], 21.2% of women across the EU member states experienced physical and/or sexual violence by their partner, whereas in Italy the average rate is 17%. This is noteworthy considering that European States with greater gender equality show the highest levels of IPV [43]. It is still not clear whether these differences are due to the fact that women in more equal countries are more likely to report the violence or that there are other socio-cultural reasons [43]. Further research is needed to address this point. Thus, since the cultural dimension appears to play a key role in both IPV myths and moral disengagement, cross-cultural investigations should assess the replicability of these findings.

## 5. Conclusions

Taken together, our results underline the relevance of sociocultural dimensions in endorsing domestic violence myth acceptance and moral disengagement in sexual harassment. Since these phenomena represent a relevant public health issue and have a long-term impact on victim’s psychological functioning, prevention and intervention programs should also address the social and community level. It is hoped that the present data could provide useful considerations for professionals. Specifically, these findings can be used in creating programs to enhance the knowledge and awareness of violence and de-construct violence myths and harassment justification. There are several existing programs addressing these issues (see, for instance, United Nations and WHO approach, as well as the recent review by Toman and colleagues on efforts to engage men and boys in gender violence prevention) [44]. However, such programs could be improved by taking into account the crucial role of hostility toward women and benevolence toward men, as well as biological lay theories, that reinforce and enhance the support of myths and moral disengagement. Such programs should include contents aimed at eradicating these sexist attitudes.

Finally, similar considerations may inform policy recommendations in universities. Intimate partner violence and sexual harassment are particularly frequent in university settings [45], with relevant harmful consequences on mental health and academic performance. Promoting an environment capable of recognizing and then reporting episodes of abuse upholds the rights of students to fair treatment.

## Figures and Tables

**Table 1:** Gender differences on the study variables: means, standard deviations, and T-test scores.

		Mean	SD	T
BS	Men	1.79	1.21	−0.87
	Women	1.65	1.16	
HS	Men	1.72	1.26	−5.04 **
	Women	1.08	0.91	
BM	Men	1.17	0.97	−2.94 **
	Women	0.85	0.83	
HM	Men	1.75	0.82	3.09 **
	Women	2.13	0.99	
Biological theory	Men	2.65	1.08	−2.79 **
	Women	2.34	0.81	
Social theory	Men	4.18	0.98	2.79 **
	Women	4.49	0.86	
Domestic Violence Myths	Men	2.67	0.82	−4.75 **
	Women	2.23	0.68	
Moral disengagement	Men	2.74	1.09	−9.47 **
	Women	1.74	0.69	

** *p* < 0.01.

**Table 2:** Pearson’s correlations between study variables.

	1	2	3	4	5	6	7
1. BS							
2. HS	0.55 **						
3. BM	0.71 **	0.67 **					
4. HM	0.57 **	0.36 **	0.45 **				
5. Biological theory	0.51 **	0.48 **	0.55 **	0.34 **			
6. Social theory	−0.28 **	−0.32 **	−0.38 **	−0.02	−0.42 **		
7. Violence Myths	0.34 **	0.65 **	0.51 **	0.19 **	0.38 **	−0.27 **	
8. Moral disengagement	0.31 **	0.60 **	0.46 **	−0.09	0.34 **	−0.30 **	0.60 **

** *p* < 0.01

**Table 3:** Hierarchical regression analysis predicting domestic violence myth acceptance.

	Step 1	Step 2	Step 3
	β (SE)	β (SE)	β (SE)
Sex (1 = women, 2 = men)	0.22 ** (0.09)	0.05 (0.08)	0.05 (0.08)
BS		−0.10 (0.04)	−0.12 (0.04)
HS		0.56 ** (0.04)	0.55 ** (0.04)
BM		0.22 ** (0.06)	0.19 ** (0.06)
HM		−0.02 (0.04)	−0.02 (0.04)
Biological theory			0.13 * (0.05)
Social theory			−0.06 (0.00)
Adjusted R^2^	0.05	0.44 **	0.45 **
R^2^ change	0.05	0.40	0.01
F for change	16.45 **	59.38 **	2.66

** *p* < 0.01; * *p* < 0.05

**Table 4:** Hierarchical regression analysis predicting moral disengagement in sexual harassment.

	Step 1	Step 2	Step 3
	β (SE)	β (SE)	β (SE)
Sex (1 = women, 2 = men)	0.45 ** (0.10)	0.29 ** (0.09)	0.29 ** (0.09)
BS		−0.01 (0.05)	−0.03 (0.05)
HS		0.48 ** (0.05)	0.46 ** (0.05)
BM		0.13 (0.07)	0.12 (0.07)
HM		−0.10 (0.05)	−0.08 (0.05)
Biological theory			0.02 (0.05)
Social theory			−0.07 (0.05)
Adjusted R^2^	0.20 **	0.47 **	0.47 **
R^2^ change	0.20	0.28	0.00
F for change	82.77 **	43.08 **	1.72

** *p* < 0.01
